# Structural requirements of *Holothuria floridana* fucosylated chondroitin sulfate oligosaccharides in anti-SARS-CoV-2 and anticoagulant activities

**DOI:** 10.1371/journal.pone.0285539

**Published:** 2023-05-11

**Authors:** Marwa Farrag, Rohini Dwivedi, Poonam Sharma, Deepak Kumar, Ritesh Tandon, Vitor H. Pomin

**Affiliations:** 1 Department of BioMolecular Sciences, University of Mississippi, Oxford, Mississippi, United States of America; 2 Department of Pharmacognosy, Faculty of Pharmacy, Assiut University, Assiut, Egypt; 3 Center for Immunology and Microbial Research, Department of Cell and Molecular Biology, University of Mississippi Medical Center, Jackson, Mississippi, United States of America; 4 Department of Medicine, University of Mississippi Medical Center, Jackson, Mississippi, United States of America; 5 Research Institute of Pharmaceutical Sciences, School of Pharmacy, University of Mississippi, Oxford, MS, United States of America; Bangor University, SWEDEN

## Abstract

Fucosylated chondroitin sulfate (FucCS) is a unique glycosaminoglycan found primarily in sea cucumbers. This marine sulfated glycan is composed of a chondroitin sulfate backbone decorated with fucosyl branches attached to the glucuronic acid. FucCS exhibits potential biological actions including inhibition of blood clotting and severe acute respiratory syndrome coronavirus (SARS-CoV-2) infection. These biological effects have been attributed to certain structural features, including molecular weight (MW), and/or those related to fucosylation, such as degrees of fucosyl branches, sulfation patterns and contents. In a previous work, we were able to generate oligosaccharides of the FucCS from *Pentacta pygmaea* (PpFucCS) with reduced anticoagulant effect but still retaining significant anti-SARS-CoV-2 activity against the delta strain. In this work, we extended our study to the FucCS extracted from the species *Holothuria floridana* (HfFucCS). The oligosaccharides were prepared by free-radical depolymerization of the HfFucCS via copper-based Fenton reaction. One-dimensional ^1^H nuclear magnetic resonance spectra were employed in structural analysis. Activated partial thromboplastin time and assays using protease (factors Xa and IIa) and serine protease inhibitors (antithrombin, and heparin cofactor II) in the presence of the sulfated carbohydrates were used to monitor anticoagulation. Anti-SARS-CoV-2 effects were measured using the concentration–response inhibitory curves of HEK-293T-human angiotensin-converting enzyme-2 cells infected with a baculovirus pseudotyped SARS-CoV-2 wild-type and delta variant spike (S)-proteins. Furthermore, the cytotoxicity of native HfFucCS and its oligosaccharides was also assessed. Like for PpFucCS, we were able to generate a HfFucCS oligosaccharide fraction devoid of high anticoagulant effect but still retaining considerable anti-SARS-CoV-2 actions against both variants. However, compared to the oligosaccharide fraction derived from PpFucCS, the average MW of the shortest active HfFucCS oligosaccharide fraction was significantly lower. This finding suggests that the specific structural feature in HfFucCS, the branching 3,4-di-sulfated fucoses together with the backbone 4,6-di-sulfated N-acetylgalactosamines, is relevant for the anti-SARS-CoV-2 activity of FucCS molecules.

## Introduction

The coronavirus disease 2019 (COVID-19), caused by severe acute respiratory syndrome coronavirus 2 (SARS-CoV-2), recently provoked a pandemic of vast catastrophic consequences in the world [1]. Since the onset of the outbreak with the wild-type (Wuhan-HU-1) SARS-CoV-2 strain in late 2019 in Wuhan, China, SARS-CoV-2 variants of concern (VOC) have been emerging [2]. This phenomenon has been driven primarily by the high-rate mutations encountered during the process of virus evolution and spread. Unfortunately, a series of health threats have appeared along with the VOC evolution, including but not limited to increased transmissibility, virulence, reduced vaccine effectiveness, and altered antigenicity [3]. The delta (B.1.617.2) variant has been included among the most transmissible and virulent VOCs that have recently appeared in our society [4].

Mechanistically speaking, molecular interactions made by the spike (S)-protein, an essential structural protein found on the envelope surface of SARS-CoV-2, are formed during the initial stage of the viral infection. In this stage, S-protein recognizes and interacts specifically with the host cell receptor angiotensin-converting enzyme 2 (ACE2) [5]. In addition to ACE2, the S-protein must also interact with heparan sulfate (HS) proteoglycans, which act as a cell co-receptor. Hence, S-protein interactions with these two different molecules at the surface of the host cells are essential for virus adhesion and invasion during the SARS-CoV-2 infection [5].

**Fig 1:**
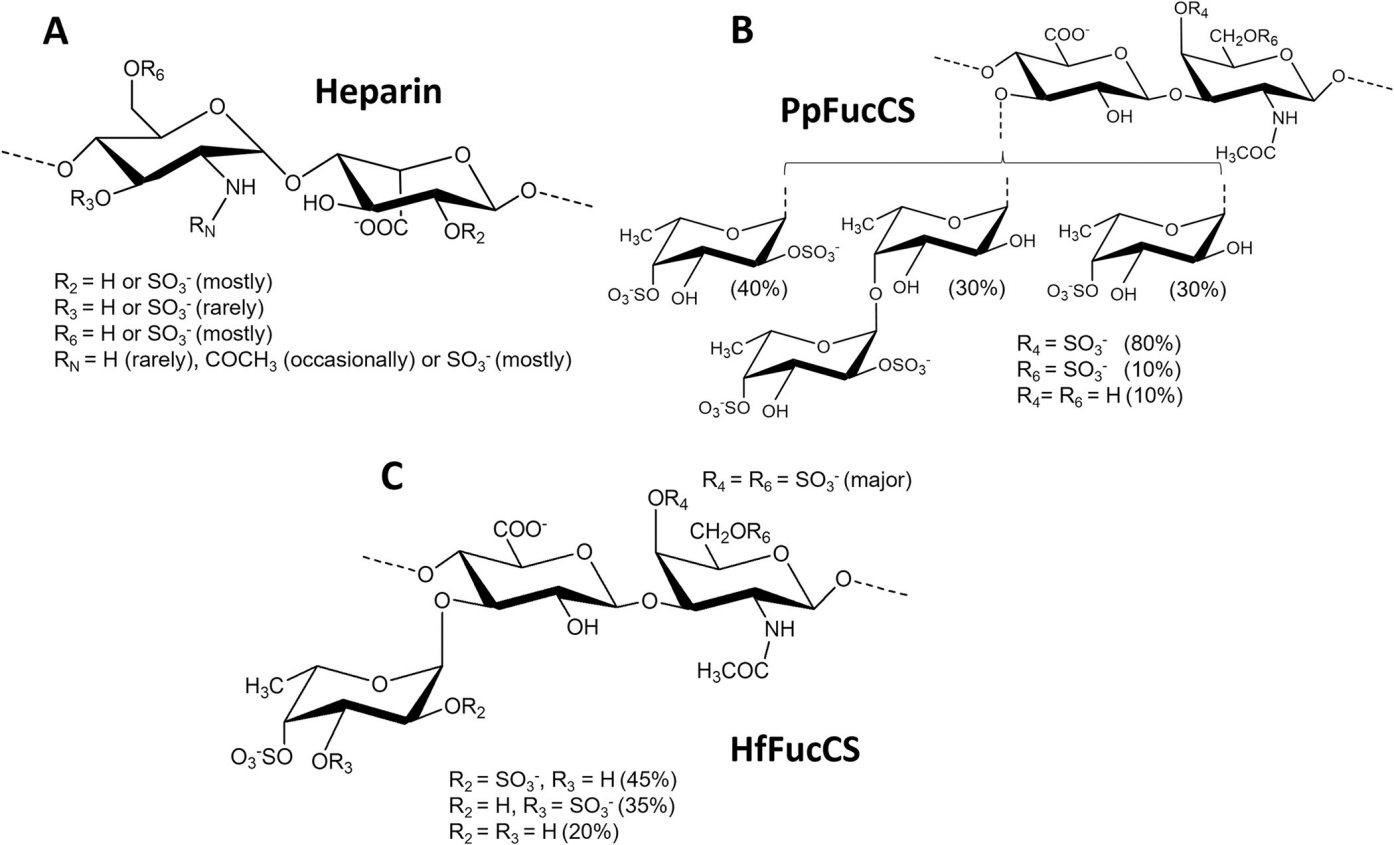
Structural representations of (A) heparin, (B) PpFucCS, and (C) HfFucCS. (A) Heparin is mostly composed of alternating α-GlcN and α-IdoA units in repeating disaccharide building blocks of [→4)-GlcN-(1→4)-IdoA-(1→], and sulfation occurs mostly at the C6 and N-positions of GlcN and C2 position of IdoA. However, rare substitutions like sulfation at the C3 position and acetylation at the N-position of the GlcN unit as well as non-sulfation and non-acetylation can also occur. (B) PpFucCS is composed of repeating trisaccharide and tetrasacharride units of {→3)-β-GalNAcX-(1→4)-β-GlcA-[Y(1→3)]-(1→}, where X = 4S (80%), 6S (10%) or 0S (10%) and Y = α-Fuc-2,4S, α-Fuc-2,4S-(1→4)-α-Fuc, or α-Fuc4S in a ratio of 40:30:30. (C) HfFucCS is composed of repeating trisaccharide units {→3)-β-GalNAcX-(1→4)-β-GlcA-[Y(1→3)]-(1→}, where X = 4,6S (majorly) and Y = α-Fuc-2,4S, α-Fuc-3,4S, or α-Fuc-4S in a ratio of 45:35:20. IdoA, GlcN, GlcNS, GalNAc, GlcA, Fuc, and S represent L-iduronic acid, D-glucosamine, N-sulfated D-glucosamine, N-acetyl D-galactosamine, D-glucuronic acid, L-fucose, and sulfate (SO_3_^-^), respectively.

By administering an exogeneous inhibitor or competitor, an intentional disruption of the molecular interactions between the viral S-proteins with one of these molecules can be achieved leading therefore to an anti-SARS-CoV-2 outcome. Indeed, key studies have recently demonstrated the anti-SARS-CoV-2 capacity of numerous molecules, including ACE2 inhibitors, heparin (Fig 1A), and other sulfated glycans of various structures and sources [6–14]. Furthermore, different levels of anti-SARS-CoV-2 activity have been achieved, and this differential activity has been in turn attributed to the overall or specific structural features of the administered molecules.

For the case of sulfated glycans studied as antagonists of HS, for instance, one clinical challenge exists in the development of these molecules as promising candidates against SARS-CoV-2. The challenge comes from the fact that this class of molecules, e.g., heparin (Fig 1A), are endowed with potential anticoagulant effects, which might pose bleeding or hemorrhagic risks during their clinical administration as antivirals [15–18]. Recently, however, we were able to present an efficient strategy to increase selectivity of action by dissociating the anti-SARS-CoV-2 and anticoagulant activities of two different marine sulfated glycans (MSGs): one sulfated galactan from the red alga, *Botryocladia occidentalis* [12], and one fucosylated chondroitin sulfate (FucCS) from the sea cucumber, *Pentacta pygmaea* (PpFucCS) (Fig 1B) [19]. This strategy relies on a chemical depolymerization process of these MSGs using a controlled hydrolysis. Since the anticoagulant activity of MSGs are known to be generally molecular weight (MW)-dependent [20, 21], oligosaccharides of lower MWs than the native molecules can eventually lead to reduced anticoagulant properties with little or no impact on the potency or efficacy of the antiviral response [12, 19]. Since each MSG has its own structure and can present different levels of anticoagulant versus anti-SARS-CoV-2 activities, we believe that each MSG can respond differently to the applied chemical strategy of depolymerization for segregation of the two biological activities.

Following this rationale, we have successfully extended our strategy to the FucCS extracted from the sea cucumber, *Holothuria floridana* (HfFucCS) (Fig 1C) [22]. Oligosaccharides were prepared via a copper-based Fenton reaction, which enables chemical cleavage only on the chondroitin sulfate backbone of FucCS and not on the fucosyl branching sites or on the chemical bonds of the sulfate groups [18, 19, 23–26]. The structural integrity of the formed HfFucCS oligosaccharides were then verified using one-dimensional (1D) ^1^H nuclear magnetic resonance (NMR) spectroscopy. Their anticoagulant effects were measured using activated partial thromboplastin time (aPTT), and inhibitory assays using protease (factors Xa and IIa) and serine protease inhibitors (antithrombin [AT] and heparin cofactor II [HCII]) in the presence of the exogenous polysaccharides. Anti-SARS-CoV-2 properties were measured using HEK-293T-human ACE2 (HEK-293T-hACE2) cells infected with a baculovirus pseudotyped SARS-CoV-2 wild-type (Wuhan-Hu-1) and delta (B.1.617.2) spike (S)-protein variants. Cytotoxicity, which was assessed against the HEK-293T-hACE2 cells, showed no harmful effects of HfFucCS or any of its oligosaccharide preparations. Similar with previously published results about PpFucCS, we were also able to generate HfFucCS oligosaccharides with retained anti-SARS-CoV-2 activities against both wild type and delta, and devoid of high anticoagulant effects. Curiously, however, the oligosaccharides generated in this work had significantly shorter chain lengths than those of the antivirally active PpFucCS oligosaccharides. A structural comparison between PpFucCS and HfFucCS (Fig 1B versus 1C) enabled us to indicate the branching 3,4-di-sulfated fucose (Fuc) units together with the backbone 4,6-di-sulfated N-acetylgalactosamine (GalNAc) units of HfFucCS as a potential active motif in FucCS molecules for their anti-SARS-CoV-2 activity. Although HfFucCS has already been structurally characterized and reported [22], and Fenton reaction has already been exploited for depolymerization of FucCS molecules [18, 19, 23–26], this is the first report applying HfFucCS oligosaccharides produced by this method in investigations regarding both anti-SARS-CoV-2 and anticoagulant actions to identify potential key motifs responsible for the activity.

## Results and discussion

### Extraction, purification, and physicochemical analyses of HfFucCS

The crude polysaccharide extracted from the body wall of the sea cucumber, *H*. *floridana*, was subjected to an anion-exchange chromatography on a DEAE Sephacel column (S1 Fig). Two peaks were eluted with a linear NaCl gradient performed from 0 to 3 M concentration: the fucosylated chondroitin sulfate (HfFucCS) eluted at 1.2 M NaCl and a sulfated fucan (HfSF) eluted at 1.7 M NaCl. Both HfFucCS and HfSF have previously been structurally characterized and biologically studied as potential inhibitors of heparinase, coagulation, and the delta variant of SARS-CoV-2 [14, 22, 27]. Oligosaccharides derived from HfSF have been produced and structurally investigated in a previous work [28], whereas those derived from HfFucCS have virtually never been investigated in anticoagulation and as anti-SARS-CoV-2 agents. Therefore, in this work, we decided to fill up this gap of the scientific literature.

**Fig 2:**
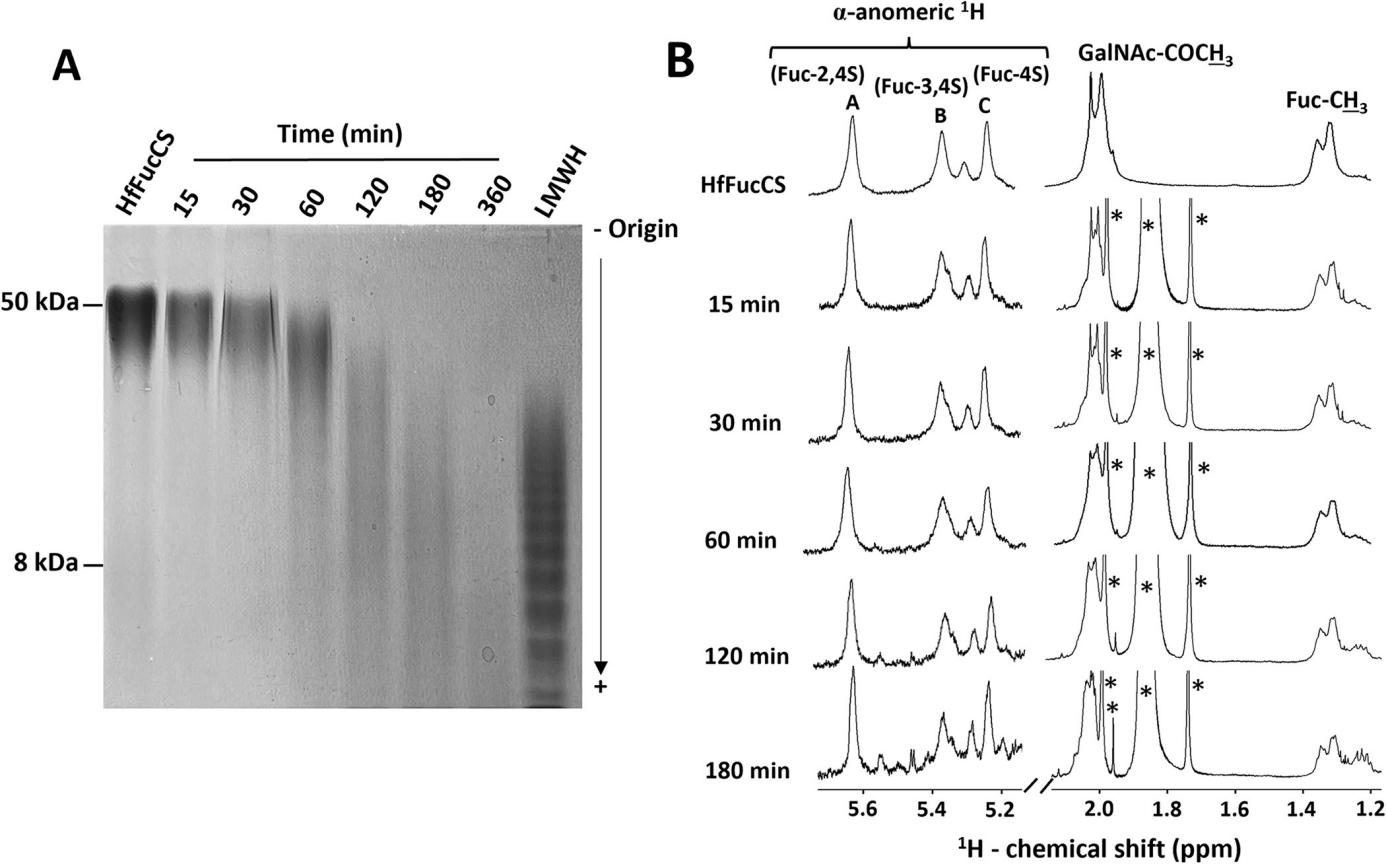
Physicochemical assessment of native and hydrolyzed fractions of HfFucCS using (A) PAGE and (B) 1D ^1^H NMR spectroscopy. (A) The native HfFucCS (first lane) and depolymerized products obtained within different time courses (15, 30, 60, 120, 180, and 360 minutes) of free-radical Fenton-based depolymerization reaction (2 mg/ml of HfFucCS hydrolyzed with 0.02 mM copper (II) acetate and 100 mM hydrogen peroxide) were analyzed using PAGE. Samples (10 μg each) were loaded on a 22% polyacrylamide gel and stained with 0.1% (w/v) toluidine blue (in 1% acetic acid) after adequate electrophoretic migration. The molecular markers used included LMWH (~8 kDa) and the native HfFucCS (~50 kDa). (B) 1D ^1^H NMR spectral stack of native HfFucCS (top) and depolymerized products was obtained within different time courses (15, 30, 60, 120, 180, and 360 minutes). The α-anomeric proton (^1^H1) signals from the three differently sulfated Fuc branches are shown on the left low-field region with δ_H_ expansion of 5.7–5.1 ppm. The signals are labeled as A (α-Fuc-2,4S), B (α-Fuc-3,4S), and C (α-Fuc-4S). Diagnostic ^1^H signals from the methyl protons of GalNAc-COCH_3_ and of Fuc-CH_3_ are shown on the right up-field region with δ_H_ expansion of 2.2–1.2 ppm. 1D ^1^H NMR spectra were recorded in D_2_O, at 50°C, on a 500 MHz Bruker NMR instrument. The signals indicated with asterisks are from the solvent and/or reagents used in the depolymerization reaction.

HfFucCS is structurally composed of repeating trisaccharide units with a chondroitin sulfate (CS) type-E (CS-E) backbone, characteristic of 4,6-di-sulfation in 3-linked β-N-acetylgalactosamine (GalNAc) units, decorated with α- Fuc branches linked at the C3 position of the backbone 4-linked β-glucuronic acid (GlcA) unit (Fig 1C). The Fuc branches are differently sulfated, with a ratio of 45:35:20 for 2,4-di-sulfated Fuc (α-Fuc-2,4S), 3,4-di-sulfated Fuc (α-Fuc-3,4S), and 4-sulfated Fuc (α-Fuc-4S). The average MW of HfFucCS has been previously suggested as ~50 kDa [22], and the current analysis based on polyacrylamide gel electrophoresis (PAGE) indicates electrophoretic migration roughly around this MW range, as noted from the single band in the middle of the top half of the gel, significantly above migration of the low MW heparin (LMWH) of average MW of ~8 kDa, and significantly below the origin of the gel (≥ 100 kDa) (Fig 2A). 1D ^1^H NMR spectrum of HfFucCS has been reported and correlated to the structure of Fig 1C [22]. The 1D ^1^H NMR method is significantly useful and reliable in quick assessment of multiple aspects of our HfFucCS preparation, such as (1) structural integrity of the holothurian glycan, (2) purity level of our preparation, and (3) potential chemical changes in HfFucCS upon free-radical depolymerization during oligosaccharide production (Fig 2B). Diagnostic 1D ^1^H NMR signals can be assigned to recognize the different composing monosaccharides within the native HfFucCS molecule (top spectrum at Fig 2B). The low-field region of ^1^H chemical shift (δ_H_) within the expansion of 5.65–5.20 ppm is diagnostic of the anomeric ^1^H1 signals from the differently sulfated α-Fuc units: α-Fuc-2,4S (labeled as A unit) with δ_H1_ at 5.64 ppm, α-Fuc-3,4S (labeled as B unit) with δ_H1_ at 5.35 ppm, and α-Fuc-4S (labeled as C unit) with δ_H1_ at 5.22 ppm (top spectrum at Fig 2B). The ^1^H NMR signals at the up-field region (δ_H_ expansion of 2.2–1.2 ppm) are characteristic of the methyl protons (CH_3_) of both the GalNAc and of Fuc units.

### Production of HfFucCS oligosaccharides by controlled Fenton-based reaction

Since the structure of the FucCS molecules can vary among the different holothurian species, optimal conditions must be initially established to control the depolymerization process for the generation of oligosaccharides with reasonable sizes. Hence, multiple reaction conditions were employed and analyzed using PAGE in the Fenton-based hydrolysis of HfFucCS (S2 Fig). Varying levels of HfFucCS depolymerization were achieved within different reaction time courses (15, 30, 60, 180, and 360 minutes) using three concentrations (20, 100, or 200 mM) of hydrogen peroxide (H_2_O_2_) and three concentrations (0.02, 0.2, or 2 mM) of copper (II) acetate (Cu[OAC]_2_). Temperature (60°C) and pH (6.0) were kept constant as successfully employed in our previous work with PpFucCS [19]. As expected, the increasing concentrations of H_2_O_2_, Cu(OAC)_2_ and time led to more degradation of HfFucCS (S2 Fig), and as discussed below, the decision about the best set of reaction conditions for large-scale production of HfFucCS oligosaccharides with adequate chain lengths for further fractionation could only be reached through this initial optimization study of the reaction conditions.

One of the best sets of controlled conditions observed during the optimization study was that using 2 mg HfFucCS (in 0.1 M NaOAc, pH 6.0) treated with 100 mM H_2_O_2_ in the presence of 0.02 mM Cu(OAc)_2_ at 60°C within different time courses, as highlighted in Fig 2A. Similar conditions were previously observed for depolymerization of PpFucCS, except the use of 200 mM concentration of H_2_O_2_ [19]. We then employed 1D ^1^H NMR spectra to quickly assess the structural details of the HfFucCS products at this condition, to ensure that no additional chemical changes besides only the backbone cleavage for reduction of MW, as assessed by PAGE, were occurring on HfFucCS when subjected to the Fenton hydrolysis (Fig 2B). The same ^1^H NMR profile (based on both relative peak areas and δ_H_ values) of the native HfFucCS was observed for the degraded products obtained within the different time courses, except for the appearance of additional peaks in the up-field regions, as indicated with the asterisks. These 1H signals are not from the HfFucCS structure, but from the reagents used during the Fenton-based depolymerization of this MSG. From the 1D ^1^H NMR analysis, we safely ensured that desulfation and defucosylation did not occur during the Fenton-based degradation of HfFucCS in the condition used, and from this set of hydrolysis condition, the duration of 180 minutes showed the capacity to produce oligosaccharides with similar oligosaccharide range of the LMWH (Fig 2A). Oligosaccharides can be successfully fractionated from MSGs with similar MW distribution of LMWH via size-exclusion chromatography (SEC) using Bio-Gel P-10 column, as previously reported [12, 19]. Hence, the reaction condition using 100 mM H_2_O_2_, 0.02 mM Cu(OAc)_2_, pH 6.0, at 60°C, for 180 minutes was chosen for scaled-up production of HfFucCS oligosaccharides for further fractionation.

### Size-fractionation and physicochemical analyses of HfFucCS oligosaccharides

**Fig 3:**
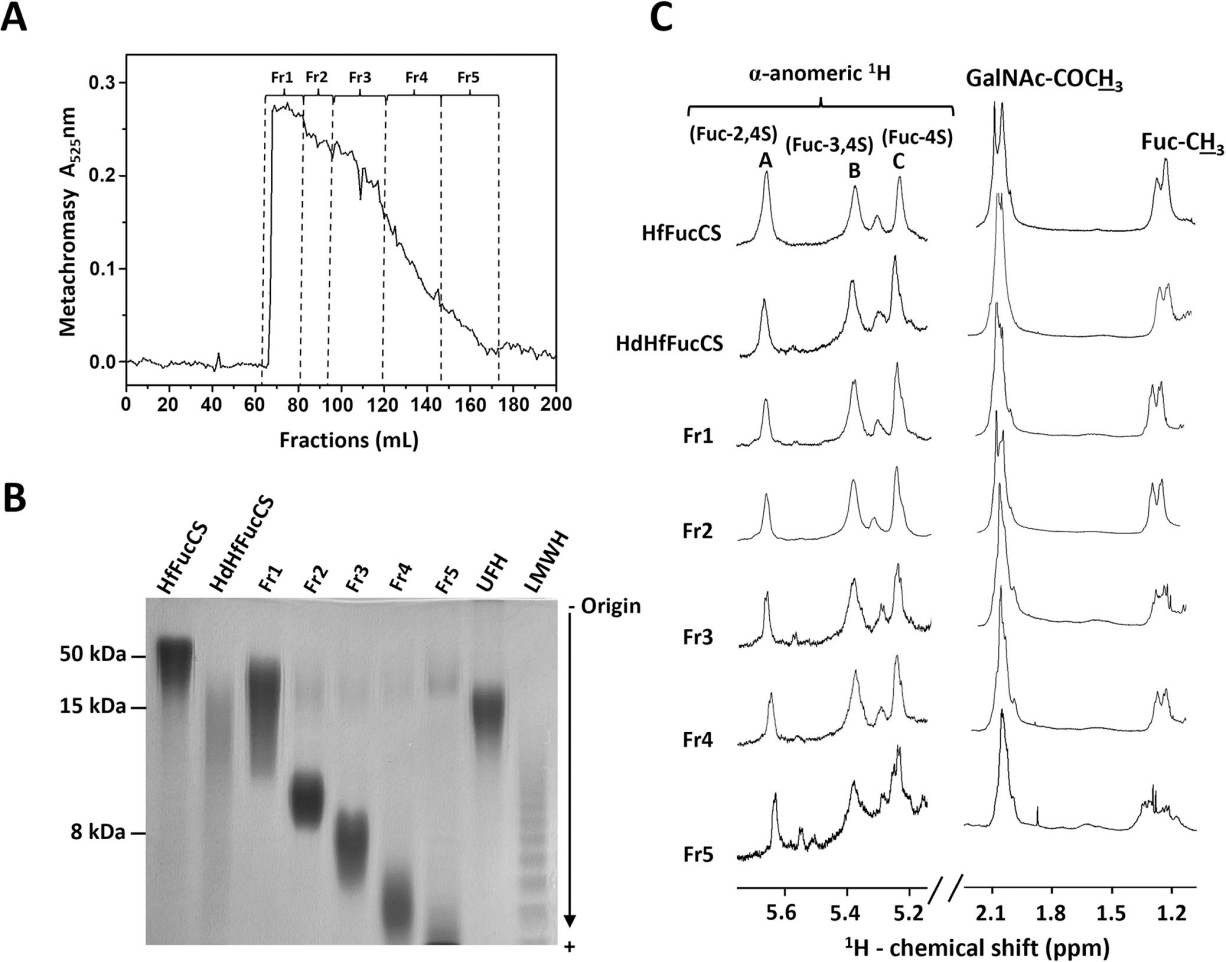
(A) Fractionation and (B and C) physicochemical analyses of HfFucCS oligosaccharides using (A) SEC Bio-Gel P-10 column, (B) PAGE, and (C) 1D ^1^H NMR spectroscopy. Around 45 mg of HdHfFucCS obtained by free-radical depolymerization using 0.02 mM copper acetate, 100 mM H_2_O_2_ (pH 6.0) for 180 minutes at 60°C were fractionated in a Bio-Gel P-10 column (1 x 120 cm). The fractions were eluted using 10% ethanol in 1M NaCl at a flow rate of 1.0 ml/15 min, and the eluted fractions were monitored by metachromatic assay using DMB (Absorbance at 525 nm). Fractions Fr1–Fr5 were randomly pooled, as indicated in the panel, and desalted on Sephadex G15 (1 x 30 cm) column, pooled, and lyophilized before further analyses. (B) HfFucCS, HdHfFucCS, and the purified HfFucCS oligosaccharides (Fr1–Fr5) were loaded into a 22% polyacrylamide gel and electrophoretic mobility was compared with the molecular markers of known MWs such as LMWH (~8 kDa) and UFH (~15 kDa). The compounds were stained with 0.1% (w/v) toluidine blue (in 1% acetic acid) after electrophoretic migration, and (C) 1D ^1^H NMR spectral stack shows the resultant spectrum of each HfFucCS sample. The α-anomeric region (δ_H_ expansion ~5.7–5.2 ppm) displays the ^1^H1 resonance of the three fucosyl branched types, α-Fuc-2,4S, α-Fuc-3,4S and α-Fuc-4S, labeled respectively as A, B, and C on the top. The methyl of GalNAc-COCH_3_ and of Fuc-CH_3_ are seen at the up-field region (δ_H_ expansion ~2.2–1.2 ppm). All 1D ^1^H NMR spectra were acquired in D_2_O at 50°C on a 500 MHz Bruker NMR instrument.

Around 45 mg of HfFucCS was depolymerized using the selected condition, and the mixture of HfFucCS oligosaccharides, namely HdHfFucCS, was fractionated by SEC in a Bio-Gel P-10 column, as described in Materials and methods. The fractions were monitored with metachromasy using the 1,9-dimethylmethyene blue (DMB) dye, and the resultant chromatogram (Fig 3A) showed a profile with no distinctive peaks. This was expected, based on the electrophoretic migration pattern observed in PAGE, with no clear presence of defined or narrow bands (sixth lane at Fig 2A, and second lane at Fig 3B). Hence, the fractionation was randomly pooled into five major fractions (Fr1–Fr5), as displayed in Fig 3A. These oligosaccharide fractions were further analyzed using PAGE (Fig 3B) and compared with LMWH (~8 kDa), unfractionated heparin (UFH) (~15kDa), native HfFucCS (~ 50 kDa), and the unfractionated mixture of 180 min-hydrolyzed HdHfFucCS sample. As expected, a gradual decrease in MW range was seen from Fr1 to Fr5, based on SEC. Fr1 showed the least reduction in MW as seen from its electrophoretic migration above the LMWH, Fr2 and Fr3 showed respectively MW distributions falling approximately on the top half and middle regions of the LMWH electrophoretic migration, while Fr4 and Fr5 were on the bottom half of the LMWH electrophoretic migration (Fig 3B). SEC-multi-angle light scattering (MALS) analyses were conducted on the HfFucCS oligosaccharide fractions (Fr1–Fr5). Results have indicated the average MWs (kDa) of HfFucCS Fr1–Fr5 respectively as 27.0 (± 6.0%), 7.4 (± 17.2%), 3.2 (± 13.4%), 1.8 (± 6.5%), and 1.2 (± 14.4%). These SEC-MALS-calculated MWs agree very well with the electrophoretic migration observed in PAGE (Fig 3B). The 1D ^1^H NMR spectral profile of the HfFucCS-derived low MW products (HdHfFucCS and Fr1–Fr5) showed the characteristic ^1^H resonances of all three differently sulfated fucosyl branches (α-Fuc-2,4S, α-Fuc-3,4S, and α-Fuc-4S) and methyl ^1^H signals from GalNAc and Fuc units with similar relative intensities (peak integrals) and same resonance frequencies (δ_H_) (Fig 3C). This set of results indicate that the fractionated oligosaccharides from HfFucCS (Fr1–Fr5) are structurally identical to the native HfFucCS molecule as seen by the similar NMR spectra (Fig 3C), despite the different (reduced) chain lengths as seen in PAGE (Fig 3B).

### Anti-SARS-CoV-2 activity and cytotoxicity of HfFucCS and oligosaccharides

**Fig 4:**
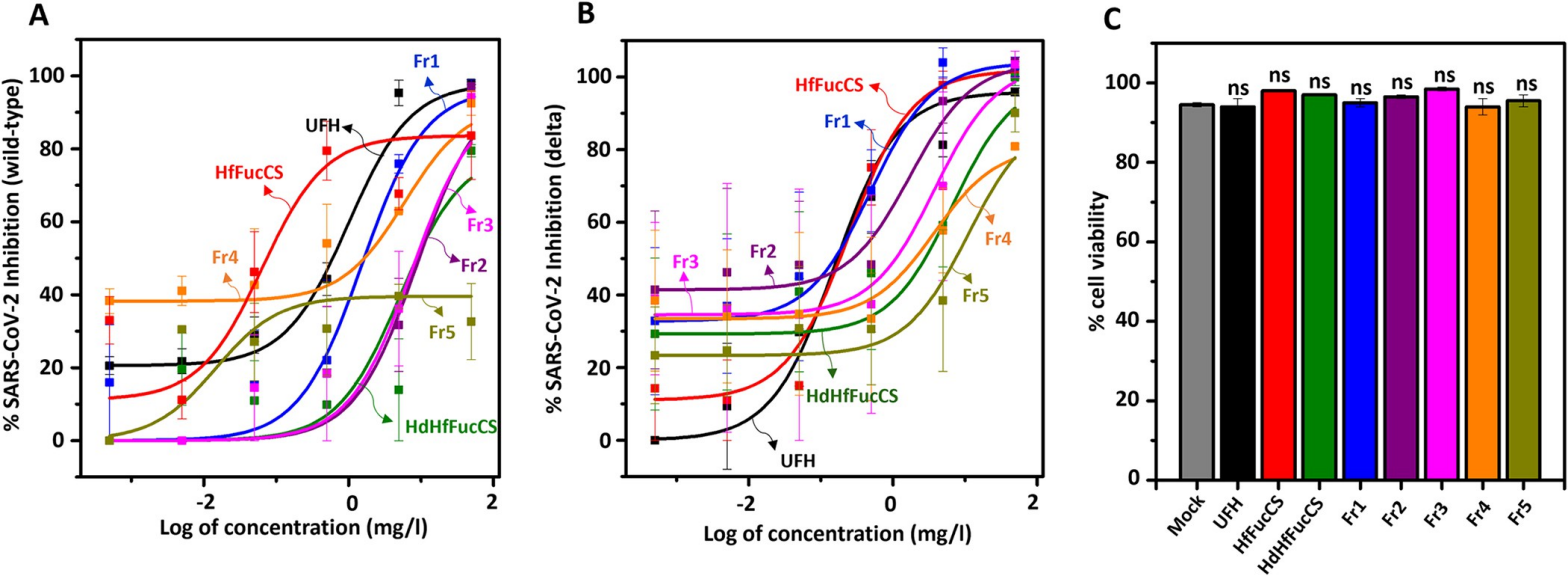
Anti-SARS-CoV-2 activity against (A) wild-type (Wuhan-Hu-1) and (B) delta (B.1.617.2) variants and (C) cytotoxicity of UFH, HfFucCS, and HfFucCS oligosaccharides. Sulfated glycans were assayed for their potential to inhibit GFP transduction in HEK-293T-hACE2 cells infected with pseudotyped SARS-Cov-2 S-protein (A) wild-type and (B) delta strains in a log-concentration-dependent manner. Non-linear regression was used to fit a dose–response curve using the least squares method for the normalized values from the assay. Each individual point represents triplicate values. Curves in the plot represent the following: UFH (black), HfFucCS (red), HdHfFucCS (olive), Fr1 (blue), Fr2 (purple), Fr3 (magenta), Fr4 (orange), and Fr5 (dark yellow). (C) Bar plots represent viability percentages of HEK-293T-hACE2 cells when treated with the sulfated glycans at the highest tested concentration (50 mg/l).

We measured the anti-SARS-CoV-2 activity of native HfFucCS and its oligosaccharides, in comparison with UFH against both wild-type (Wuhan-Hu-1) and delta (B.1.617.2) variants using a cell-based assay (Fig 4A for wild type, and Fig 4B for delta). The assay was conducted by monitoring the reduction in green fluorescent protein (GFP) expression in HEK-293T-hACE2 cells infected with baculovirus pseudotyped with SARS-CoV-2 S-proteins of either one of the variants. The half-maximal inhibitory concentration (IC_50_) values obtained from the resultant inhibitory concentration-response curves (Fig 4A and 4B) are shown in Table 1.

**Table 1:** IC_50_ values of anti-SARS-CoV-2 activities against wild-type and delta variants and anticoagulant activities of UFH, HfFucCS and HfFucCS oligosaccharides.

Sulfated glycan	Anti-SARS-CoV-2 activity				Anticoagulant activity			
	Wild type (Wuhan-Hu-1)^a^		Delta (B.1.617.2)^b^		aPTT (IU/mg)^c^ ± SE	AT/IIa	AT/Xa	HCII/IIa
	IC_50_ (μg/ml) ± SE	I_max_± SE	IC_50_ (μg/ml) ± SE	I_max_± SE		IC_50_ (μg/ml) ± SE		
**UFH**	0.60 ± 0.03	96 ± 1.12	0.17 ± 0.03	98 ± 0.22	180 ± 0.0001	0.02 ± 0.0001	0.03 ± 0.0005	0.7 ± 0.01
**HfFucCS**	0.08 ± 0.014	83 ±12.25	0.18 ± 0.06	102 ± 2.35	43.42 ± 0.25	4.67 ± 0.2	2.86 ± 0.015	0.3 ± 0.04
**HdHfFucCS**	8.13 ± 4.71	79 ± 1.65	2.80 ± 1.26	100 ± 2.30	15.00 ± 0.62	ND	52.70 ± 1.4	ND^d^
**Fr1**	1.58 ± 0.76	97 ± 2.28	0.16 ± 0.06	103 ± 2.66	40.42 ± 1.76	ND	11.75 ± 0.65	ND
**Fr2**	8.91 ± 3.56	97 ± 0.35	0.28 ± 0.09	104 ± 1.33	18.30 ± 0.79	ND	82.10 ± 3.3	ND
**Fr3**	8.51 ± 3.40	94 ± 0.02	1.12 ± 0.68	103 ± 3.65	10.34 ± 0.07	ND	54.55 ± 1.15	ND
**Fr4**	5.62 ± 0.54	92 ± 3.34	1.61 ± 0.61	81 ± 0.70	5.85 ± 0.004	ND	ND	ND
**Fr5**	ND	20 ± 13.6	7.42 ± 3.85	90 ± 5.21	6.07 ± 0.40	ND	ND	ND

^a^IC_50_ values of anti-SARS-CoV-2 inhibitory activity of UFH, HfFucCS, and HfFucCS oligosaccharides were determined against HEK 293T-hACE2 cells infected with baculovirus pseudotyped with SARS-CoV-2 S-protein of the wild type (Wuhan-Hu-1).

^b^IC_50_ values of anti-SARS-CoV-2 inhibitory activity of UFH, HfFucCS and HfFucCS oligosaccharides were determined against HEK 293T-hACE2 cells infected with baculovirus pseudotyped with SARS-CoV-2 S-protein of the delta (B.1.617.2) variant.

^c^Values were calculated using a parallel UFH (180 IU/mg) standard curve.

^d^Not determined.

Data have indicated that native HfFucCS has better potency (IC_50_ of 0.08 ± 0.014 μg/ml) but slightly weaker efficacy (I_max_ of 83 ± 12.25 μg/ml) than UFH (IC_50_ of 0.60 ± 0.03 μg/ml and I_max_ of 96 ± 1.12 μg/ml) in the anti-SARS-CoV-2 assay against the wild-type variant (Fig 4A, and Table 1). This potency is remarkably decreased upon HfFucCS degradation to the unfractionated mixture, HdHfFucCS (IC_50_ of 8.13 ± 4.71 μg/ml). However, upon fractionation of HdHfFucCS, potency is substantially recovered as seen in the highest MS fragment Fr1 (IC_50_ of 1.58 ± 0.76 μg/ml), but gradually decreased as a function of MW reduction to the lower MW HfFucCS fragments, Fr2–Fr5, (IC_50_ ranging from 5.62 to 8.91 μg/ml and I_max_ ranging from 20 to 97 μg/ml). This set of results indicates that chain length plays a key role in anti-SARS-CoV-2 activity of HfFucCS against the wild-type strain. It also explains the decreased potency and efficacy of HdHfFucCS as a mixture of fractions (Fr1–Fr5) with different levels of anti-SARS-CoV-2 activity. This observation indicates that selectivity of anti-SARS-CoV-2 action against wild-type variant can be achieved by fractionation and selection of the HfFucCS oligosaccharides with highest MW, Fr1, although still not potent as the native HfFucCS (Fig 4A and Table 1).

Native HfFucCS has shown nearly equal potency (IC_50_ of 0.18 ± 0.06 μg/ml), but slightly better efficacy (I_max_ of 102 ± 2.35 μg/ml) than UFH (IC_50_ of 0.17 ± 0.03 μg/ml and I_max_ of 98 ± 0.22 μg/ml) against the delta variant, indicating therefore similar overall activity against the SARS-CoV-2 mutant (Fig 4B and Table 1). The same behavior observed for HdHfFucCS and isolated fragments against the wild type was noted for the delta variant, with the unfractionated oligosaccharide mixture (HdHfFucCS) showing significantly reduced activity as compared to the native HfFucCS, oligosaccharides of highest MW with greater activity (in this case, Fr1 and Fr2) and oligosaccharides of lowest MW showing reduced inhibitory activity. However, as opposed to the wild type, the oligosaccharide fragments of medium-sized MW like Fr2–Fr4 showed good (as for Fr2) or slightly reduced activity (approximately 10-fold less) than the native HfFucCS (Fig 4B and Table 1). This set of data indicate that despite the same MW-dependent effect, the MW reduction in the inhibitory activity of HfFucCS oligosaccharides against the delta variant is not as impactful as that for the wild-type variant. In terms of potential cytotoxicity, none of the tested samples exhibit significant harmful effects against the HEK 293T-hACE2 cells exposed at the highest concentration of 50 mg/l (Fig 4C) tested in the cell-based experiment in Fig 4A and 4B. Taking all these results together, non-toxic oligosaccharides of reduced MW still retaining significant anti-SARS-CoV-2 activity against both variants (for instance, Fr1 for wild type, and Fr1–Fr4 for delta) can be formed and isolated from the HfFucCS preparation.

### Anticoagulant activity of HfFucCS and oligosaccharides

**Fig 5:**
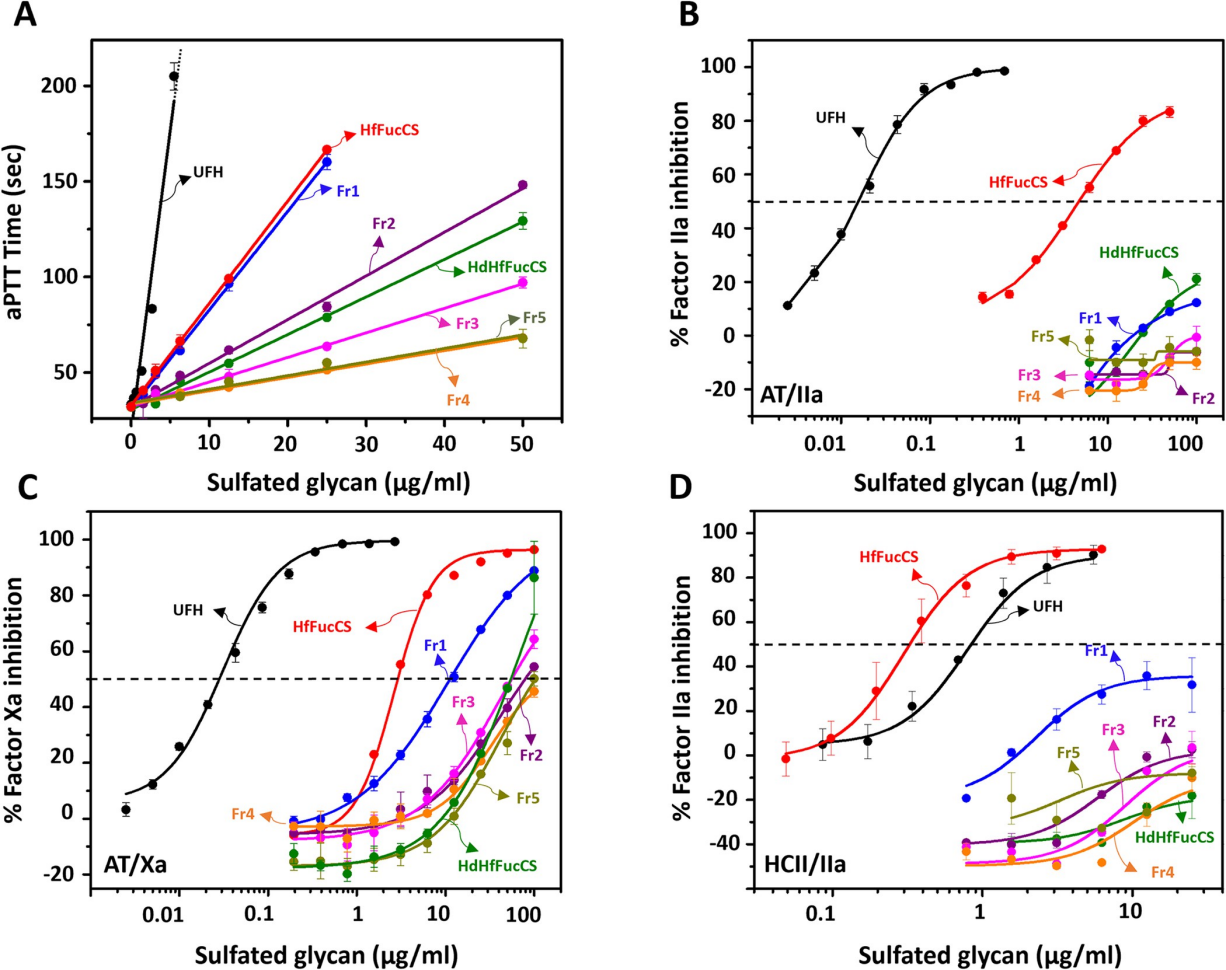
Concentration–anticoagulant response of UFH, HfFucCS, and HfFucCS-derived oligosaccharides seen by (A) aPTT, (B) AT-mediated IIa inhibition, (C) AT-mediated Xa inhibition, and (D) HCII-mediated IIa inhibition. Curves in the plots represent the following: UFH (black), HfFucCS (red), HdHfFucCS (olive), Fr1 (blue), Fr2 (purple), Fr3 (magenta), Fr4 (orange), and Fr5 (dark yellow). The dotted line in panels B–D indicate the half-maximal inhibitory activity (IC_50_ values).

The anticoagulant property of native HfFucCS, HdHfFucCS, and purified HfFucCS oligosaccharide fractions were assayed by both the aPTT method (Fig 5A) and the inhibitory assays using purified blood (co)-factors such as the proteases thrombin (IIa) (Fig 5B and 5D) and factor Xa (Fig 5C) in the presence of the serpins antithrombin (AT) (Fig 5B and 5C) and heparin cofactor II (HCII) (Fig 5D). The anticoagulant activity of the holothurian sulfated glycans were compared with standard UFH of 180 IU/mg activity. The aPTT-derived (IU/mg) and IC_50_ (μg/ml) values obtained from these experiments are shown in Table 1.

Native HfFucCS and Fr1 showed similar aPTT values of 43.4 IU/mg and 40.4 IU/mg, respectively (Fig 5A and Table 1), and the activities of these two HfFucCS molecules were markedly lower than those of the standard UFH. The aPTT values of HdHfFucCS and Fr2–Fr5 were even reduced to the values of 15.00, 18.3, 10.3, 5.8, and 6.0 IU/mg, respectively. As observed for the anti-SARS-CoV2 activities of these fragments on both strains, HdHfFucCS showed an intermediate anticoagulant action, seen by the aPTT method, somewhere between the higher activity of oligosaccharides of higher MW (Fr1 and Fr2) and the lower activity of oligosaccharides of low MW (Fr3–Fr5). This also indicates a MW-dependent phenomenon for the anticoagulant activity of HfFucCS.

This MW-dependent contribution was more evidenced in the inhibitory assays in Fig 5B–5D. Only the native showed capacity to inhibit IIa in the presence of AT (Fig 5B) and HCII (Fig 5D), with IC_50_ values respectively of 4.67 ± 0.2 and 0.3 ± 0.04 μg/ml (Table 1). The more pronounced HCII-mediated IIa inhibition than UFH is well known among the MSGs [12, 13, 15–17, 19, 20]. The hydrolyzed materials of HfFucCS were significantly inactive or inefficient in these systems, although a tendency of slightly better activity could be noted for those with the highest MW (Fig 5B and 5D). A more pronounced but still weak activity could be seen in the AT-mediated Xa inhibition (Fig 5C) in which the native HfFucCS was ~ 100-fold less active than UFH, and oligosaccharides were even weaker (IC_50_ range of 11.7 to 82.1 μg/ml) (Fig 5C and Table 1). As seen for the anti-SARS-CoV-2 activities and the aPTT, the HdHfFuCS showed an intermediate AT-mediated anti-Xa activity with IC_50_ of 52.7 ± 1.4 μg/ml, weighted by the high activity of the highest MW oligosaccharide fraction, Fr1 with IC_50_ of 11.7 ± 0.6 μg/ml, and the low activity of the oligosaccharide fractions of low MW, Fr2 and Fr3, with IC_50_ of 82.1 ± 3.3, and 54.5 ± 1.1 μg/ml, respectively. Although both biological actions, anti-SARS-CoV-2 and anticoagulation of HfFucCS are MW-dependent, the impact of MW reduction was generally more pronounced in the anticoagulant effect. This indicates that selecting a HfFucCS oligosaccharide with significantly reduced anticoagulant action but still retaining some reasonable anti-SARS-CoV-2 property is possible through the strategy of controlled Fenton-based hydrolysis and SEC fractionation.

### Comparison between the current results from HfFucCS with previously published results from PpFucCS

In a previous work, Fenton-based reaction was also used to depolymerize the PpFucCS and a low anticoagulant oligosaccharide fraction (PpFucCS Fr1) was identified with significant anti-SARS-CoV-2 effect against the delta variant [19]. This fraction however was the one with the highest MW among the four fractionated fractions (Fr1–Fr4) obtained in the work. Electrophoretic mobility of PpFucCS Fr1 showed migration on the half-top region of the standard LMWH. The PpFucCS Fr1 is analogous to the HfFucCS Fr2 in Fig 3B in terms of electrophoretic mobility, and in this work on HfFucCS, we noted that Fr4 was the shortest oligosaccharide fraction capable of retaining great anti-SARS-CoV-2 activity against the delta variant (Fig 4B and Table 1), devoid, at the same time, of high anticoagulant property (Fig 5 and Table 1). The electrophoretic mobility of HfFucCS Fr4 is below the bottom-half of the LMWH migration (Fig 3B). Compared with the PpFucCS fractions, HfFucCS Fr4 would be similar in terms of oligosaccharide distribution to PpFucCS Fr4, which was significantly inactive against delta SARS-CoV-2 and in anticoagulation [19]. This comparison of results between our previous work about PpFucCS and the current work about HfFucCS indicates that the antiviral activity of HfFucCS against the delta variant can be pushed toward significantly lower MW oligosaccharide fractions, in this case, HfFucCS Fr4. This phenomenon could only be possible if HfFucCS could present a specific structural feature, different than PpFucCS, that could enable HfFucCS to retain high anti-SARS-CoV-2 properties against the delta variant in lower MW oligosaccharides. If we compare the structures of PpFucCS (Fig 1B) and HfFucCS (Fig 1C), the 35% of branching 3,4-di-sulfated Fuc units together with the backbone GalNAc units mostly 4,6-di-sulfated in HfFucCS were evidenced as the main different features between the FucCS molecules, and based on these observations, we believe that these structural motifs are important for the anti-SARS-CoV-2 activities of FucCS molecules. To unequivocally prove this hypothesis, future works using a combination of multiple techniques such as strong anion-exchange chromatography, NMR, mass spectrometry, besides the required cell-based anti-SARS-CoV-2 assay, should be done in attempt to identify other FucCS molecules with this motif (branching 3,4-disufated Fuc and backbone 4,6-di-sulfated GalNAc units), or fractions of HfFucCS oligosaccharides rich in this motif.

## Conclusions

In a previous work, we were able to generate a high-MW oligosaccharide fraction of the PpFucCS from the holothurian species, *P*. *pygmaea*, with significant anti-SARS-CoV-2 activity against the delta strain. In this work, we extended our study to HfFucCS from the species *H*. *floridana*. As before, oligosaccharides were prepared by free-radical depolymerization via the copper-based Fenton reaction. 1D ^1^H NMR spectra were employed in structural analysis to confirm that the HfFucCS oligosaccharides showed the same structure of the starting molecule. *In vitro* anticoagulant and anti-SARS-CoV-2 assays were performed, and as previously reported for PpFucCS, we were able to generate a low anticoagulant HfFucCS oligosaccharide fraction with considerable activity against SARs-CoV-2 and no cytotoxic effect, with much lower MW than the PpFucCS. These findings suggest the specific structural feature in HfFucCS, the branching 3,4-di-sulfated Fuc together with the backbone 4,6-di-sulfated GalNAc, as a relevant motif for the anti-SARS-CoV-2 activity of FucCS molecules, especially against the delta variant.

## Materials and methods

### Extraction and purification of HfFucCS

The crude polysaccharide extract containing HfFucCS was obtained from the body wall of the sea cucumbers, *H*. *floridana* (Gulf Specimen Lab, Gulf of Mexico, Florida Keys), by enzymatic digestion using papain (1.0 mg papain/g tissue) (# P4762, Sigma-Aldrich) following a previously reported protocol [28]. The obtained crude extract was then fractionated by anion-exchange chromatography on column (2.5 x 20 cm, # Bio-Rad Laboratories) packed with DEAE Sephacel ^TM^ resin (# 17-0500-01, Cytiva, Marlborough, MA, USA) and equilibrated with 0.1 M sodium acetate buffer (pH 6.0). Elution was performed using a linear gradient of NaCl (in 0.1 M NaOAc, pH 6.0) from 0 to 3 M at a flow rate of 9 ml/10 min/fraction, applied over 24 hours. Eluted fractions were monitored by metachromatic assay using DMB reagent; absorbance was measured at 525 nm. The obtained chromatogram (S1 Fig) revealed two peaks, fractions related to each peak were pooled separately, dialyzed 3-times against water for desalting and then lyophilized. From ^1^H-NMR analysis and in accordance with a previous literature [27], the first peak was identified as HfFucCS.

### Optimization of free-radical depolymerization reaction conditions of HfFucCS

HfFucCS oligosaccharides was obtained by controlled depolymerization of HfFucCS using the modified Fenton chemistry version of an earlier reported protocol [19]. Nine different reaction conditions (S2 Fig) were performed in small scale to select the best condition for scaled-up depolymerization. Reaction conditions were optimized using varying concentrations of copper (II) acetate (0.02, 0.2, or 2 mM, final concentration) and H_2_O_2_ (20, 100, or 200 mM, final concentration). Each reaction contained HfFucCS dissolved in 0.1 M sodium acetate, pH 6.0. (2 mg/ml) with copper (II) acetate, followed by dropwise addition of H_2_O_2_. Each reaction was carried out at 60°C with continuous stirring, and for each reaction condition, aliquots of reaction mixture were taken out at six different time points: 15, 30, 60, 120, 180, and 360 minutes at which the reaction was stopped by cooling down to room temperature and by removing the copper ions from the reaction mixture using 50–100 Mesh-size Chelex resin (pre-equilibrated with 0.1 M sodium acetate buffer, pH 6.0) (Sigma, St. Louis, MO, USA), and kept on an end-to-end rotor for 2 hours. The suspension was then centrifuged at 3,000 rpm for 10 minutes, and the supernatant was then lyophilized.

Scale-up depolymerization for 45 mg of dry HfFucCS (2 mg/ml, dissolved in 0.1 M sodium acetate, pH 6.0) was achieved by adding copper (II) acetate (0.02 mM, final concentration) and dropwise addition of H_2_O_2_ (100 mM, final concentration) over 180 minutes at 60°C. The reaction mixture was then desalted on Sephadex G-10 (# G15120-10G, Sigma-Aldrich) column (1.5 × 50 cm), then lyophilized for further analysis. The degradation degree of HfFucCS at specific reaction condition and different time points were analyzed by running native PAGE in 250 mM Tris and 1.92 M glycine running buffer (pH 8.3). Samples (10 μg each) were loaded on 1.5-mm thick PAGE system having 4% stacking gel and a 22% resolving gel phase and stained with 0.1% (w/v) toluidine blue (in 1% acetic acid) after adequate electrophoretic migration. Their electrophoretic migration was compared with different markers, including LMWH (~8 kDa), and the native HfFucCS (~50 kDa).

A SEC column (Acquity BEH SEC column, 200 Å, 1.7 μm, 4.6 mm × 300 mm, Waters, MA) connected to a Dionex Ultimate 3000 ultra-high performance liquid chromatography system (Thermo Scientific, Sunnyvale, CA) was used to separate the HfFucCS oligosaccharide fractions Fr1–Fr5 by hydrodynamic volume. The eluent from the column was monitored by MALS using a DAWN HELEOS II MALS detector (Wyatt Technologies Co., Santa Barbara, CA). The MALS/refractive index detector was calibrated by polystyrene and bovine serum albumin. The samples were eluted by isocratic elution using 50 mM ammonium acetate on the SEC column at the flow rate of 0.2 ml/min. The dn/dc of the oligosaccharides was set at 0.15 ml/g. The Zimm plot was used in data fitting to obtain the Rayleigh ration for the MW calculation in the static light scattering.

### NMR spectroscopy

1D ^1^H NMR spectra were recorded using a Bruker Avance III HD 500 MHz instrument with 5 mm prodigy H/F-BBO cryoprobe. Native, depolymerized HfFucCS and the purified HfFucCS oligosaccharides were dissolved respectively in 220 or 550 μl D_2_O (99.90%) (Cambridge Isotope Laboratories, Inc. Andover, MA, USA) for 3- and 5-mm NMR tubes (VWR International, Radnor, PA, USA). Spectra were acquired at 50°C, and the acquired NMR data were further processed using TopSpin 4.0 software.

### Viral inhibition of HfFucCS oligosaccharides

The virus inhibitor screening was performed as previously described [12–14]. Human embryonic kidney cells (HEK-293T) expressing human angiotensin-converting enzyme 2 (HEK-293T-hACE2 cell line, BEI Resources #NR-52511) was plated in a 96-well tissue culture plate infected with a baculovirus pseudotyped with SARS-CoV-2 (Wuhan and delta strain) S-proteins containing a GFP reporter [Montana Molecular, #C1123G]. Virus titers were assessed under a fluorescence microscope (EVOS-FL, Thermo Fisher Scientific) by counting GFP-positive transduced cells (in a dilution) and multiplying by the dilution factor and the volume plated. Serial dilutions (50, 5, 0.5, 0.05, 0.005, 0.0005, and 0.00005 mg/l) of the test sulfated glycans were made in DMEM in triplicates to the final volume of 100 μl in each well. The controls used were UFH at 50 mg/l and mock-treated cells. The 2.5 μl of the pseudotype virus stock (2 × 10^10^ units per ml) was mixed with the test compounds, incubated for 1 hour, and then laid over HEK-293T-hACE2 cells plated in a 96-well plate along with 2 mM of sodium butyrate. The plate was then incubated for 60 hours, and the assay was read on a Cytation 5 automated fluorescence microscope after fixing with 3.7% formaldehyde. The relative IC_50_ values were calculated in Prism 9 (Graphpad Inc) by plotting normalized values from the assay against the concentrations (log) of sulfated glycans and analyzing by non-linear regression to fit a dose–response curve using the least squares method considering each replicate value as an individual point.

### Cytotoxicity of HfFucCS oligosaccharides

The cytotoxic activity of oligosaccharides was determined against HEK-293T-hACE2 cells seeded in 12-well tissue culture plates following the protocol reported earlier [12–14]. The confluent HEK-293T-hACE2 cells were treated with oligosaccharides at a final concentration of 50 mg/l along with 2 mM sodium butyrate. The treated cells were harvested after 60 hours of incubation and examined via trypan blue exclusion assay for viability. Assay readout was measured on a TC20 automated cell counter (BioRad) according to the manufacturer’s protocol.

### Anticoagulant measured by aPTT

The aPTT assay was conducted in an Amelung coagulometer KC4A (West Germany) using aPTT reagents and standard plasma purchased from Thermo Fisher Scientific (Waltham, MA, USA) using the same previously described method [29]. Plasma (90 μl) was incubated with 10 μl of a varying concentration of polysaccharide (0–50 μg) and 100 μl of aPTT reagent. After 3 minutes of incubation at 37°C, 0.25-M CaCl_2_ (100 μl) were added to the mixtures and the clotting time was recorded in seconds (sec). The results of the assay were calculated as international units/mg using a parallel standard curve based on International Heparin Standard (180 IU/mg).

### Serpin-mediated inhibitory activity against IIa and Xa by HfFucCS oligosaccharides

The sulfated glycans were assayed for their AT/HCII-mediated (effective concentration of 10 nM) inhibitory activity against IIa and Xa (effective concentration of 2 nM) in a 96-well micro-titer plate following the previously described protocol [30]. The reactant solution (100 μl) included 10 μl of tested samples, HfFucCS, HdHfFucCS, HfFucCS oligosaccharides (Fr1–Fr5), and standard heparin (180 IU/mg), as a positive control, at varying concentrations of 0–100 μg/ml, AT (40 μl of 25 nM) or HCII (40 μl of 25 nM) then IIa (50 μl of 4 nM) or Xa (50 μl of 4 nM) was added to initiate the reaction. All reagents and tested samples were prepared in TS/PEG buffer (0.02 M Tris/HCl, 0.15 M NaCl, and 1.0 mg/ml polyethylene glycol 8000, pH 7.4). The plate was then immediately incubated at 37°C for 1 minute, which was followed by the addition of 25 μl of chromogenic substrate S-2238 (Chromogenix, AB, Mondal, Sweden) for IIa or CS-11(32) (Aniara Diagnostica, West Chester, OH, USA) for factor Xa. The absorbance (Abs 405 nm) was then measured for 600 seconds at an interval of 10 seconds in a micro-plate reader. Wells without tested samples served as control and was considered 100% for IIa/Xa activity. The residual IIa/Xa activity in sample treated wells was calculated relative to that observed in the case of control wells. Calculated IC_50_ values represents mean ± standard error (SE) of the triplicate measurements. The coagulation factors Xa, IIa, AT and HCII were purchased from Hematologic Technologies. Sigma (St. Louis, MO, USA).

## Supporting information

S1 Fig Purification of *Holothuria floridana*-derived fucosylated chondroitin sulfate (HfFucCS) and its sulfated fucan (HfSF) using anion exchange chromatography. The crude polysaccharide was fractionated through a DEAE Sephacel column after proteolytic digestion of the sea cucumber body wall, followed by ethanol precipitation. The column was eluted with 100 mM sodium acetate buffer at increasing NaCl gradient from 0 to 3 M prepared in the same buffer (white circles). The fractions (1 ml each) were collected and monitored by metachromasy (absorbance at 525 nm) using a 1,9-dimethylmethylene blue (DMB) assay (black squares). The anti-SARS-CoV-2 activity of HfSF against both wild-type and delta variants was investigated and reported by Dwivedi et al (2022).
(TIF)

S2 Fig Optimization of free-radical depolymerization reaction conditions of HfFucCS achieved by copper-based Fenton method. Each 2 mg/ml of HfFucCS was depolymerized using one of the nine different reaction conditions with copper (II) acetate [(Cu(OAC)_2_] (at 0.02 mM, 0.2 mM, or 2 mM—molarity is fixed for each horizontal row) and hydrogen peroxide (H_2_O_2_) (at 20 mM, 100 mM, or 200 mM—molarity is fixed for each vertical column); at six different time points (15, 30, 60, 120, 180, and 360 min). Each reaction condition was quenched using chelex resin (50–100 mesh size). The native HfFucCS and hydrolyzed products from each reaction condition obtained within different time points were analyzed by polyacrylamide gel electrophoresis. Samples (10 μg each) were loaded on a 22% polyacrylamide gel and stained by 0.1% (w/v) toluidine blue (in 1% acetic acid) after adequate electrophoretic migration. The electrophoretic mobility was compared against two molecular markers: LMWH (~8 kDa), and the native HfFucCS (~50 kDa).
(TIF)
