# Supplementary figures and images for: Structural requirements of Holothuria floridana fucosylated chondroitin sulfate oligosaccharides in anti-SARS-CoV-2 and anticoagulant activities
Source: PLoS One. 2023 May 11;18(5):e0285539. doi: 10.1371/journal.pone.0285539 (PMC10174540; doi:10.1371/journal.pone.0285539)

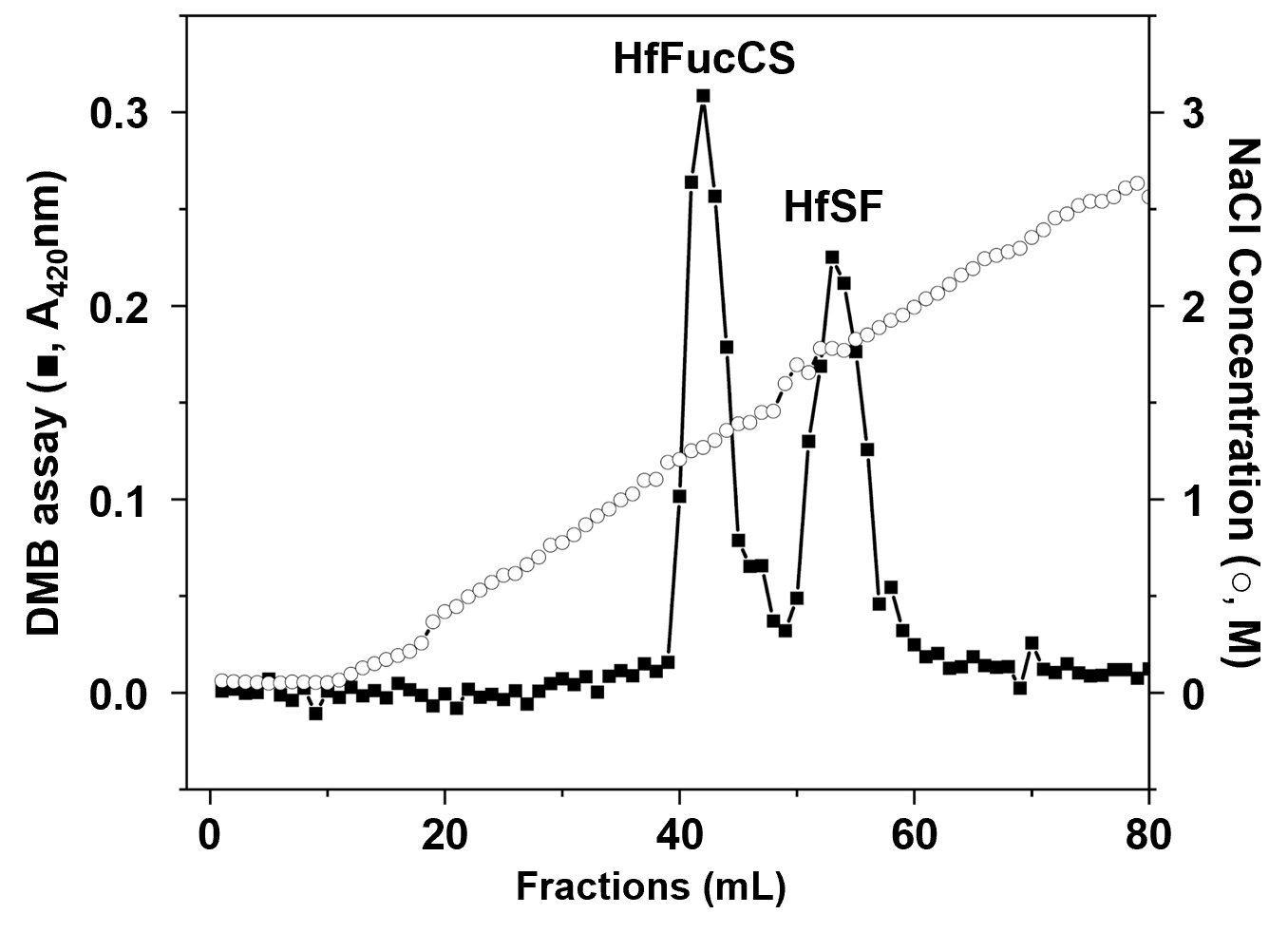

Supplement: S1 Fig — The crude polysaccharide was fractionated through a DEAE Sephacel column after proteolytic digestion of the sea cucumber body wall, followed by ethanol precipitation. The column was eluted with 100 mM sodium acetate buffer at increasing NaCl gradient from 0 to 3 M prepared in the same buffer (white circles). The fractions (1 ml each) were collected and monitored by metachromasy (absorbance at 525 nm) using a 1,9-dimethylmethylene blue (DMB) assay (black squares). The anti-SARS-CoV-2 activity of HfSF against both wild-type and delta variants was investigated and reported by Dwivedi et al (2022). (TIF) [file pone.0285539.s001.tif]

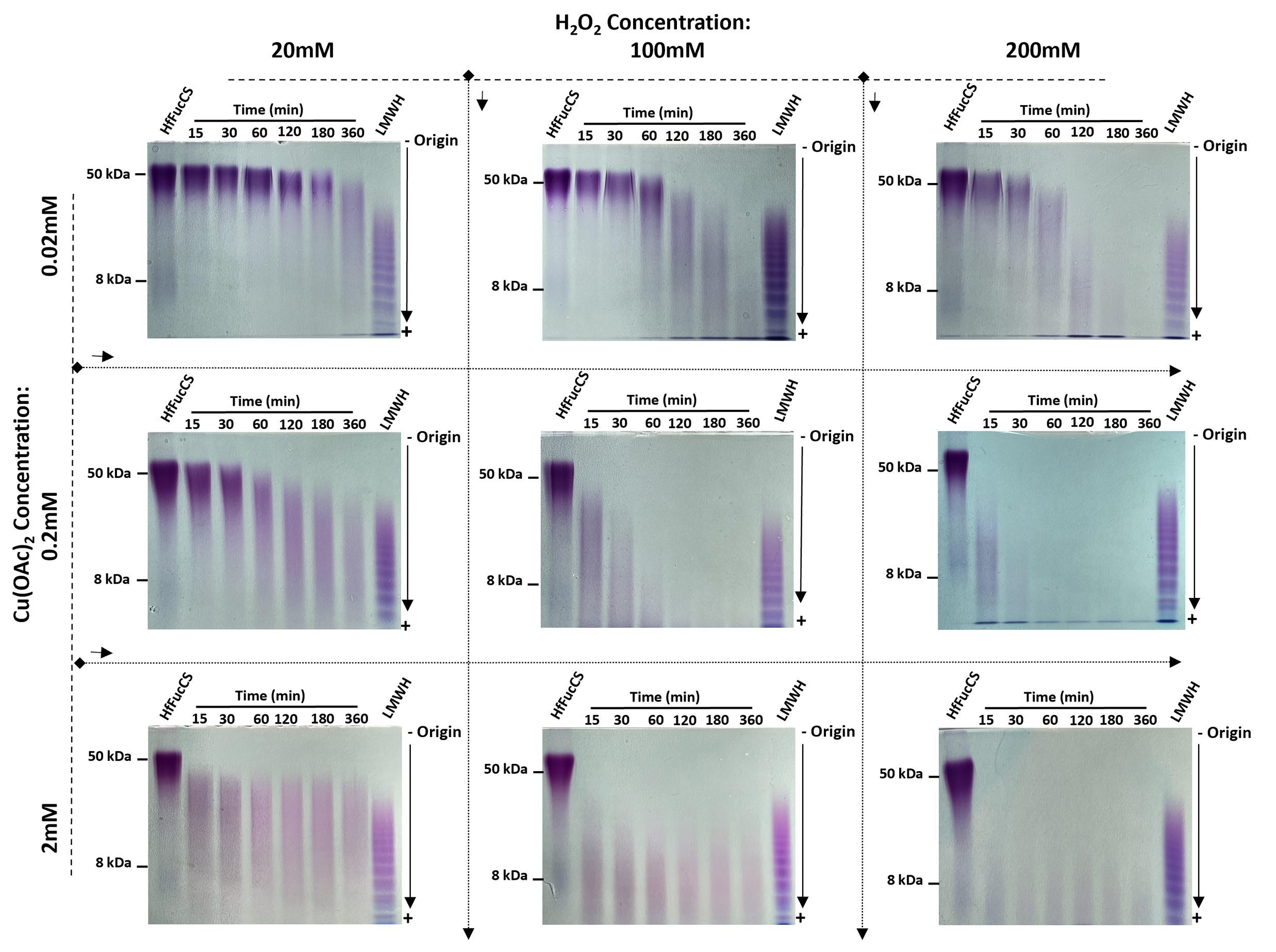

Supplement: S2 Fig — Each 2 mg/ml of HfFucCS was depolymerized using one of the nine different reaction conditions with copper (II) acetate [(Cu(OAC)2] (at 0.02 mM, 0.2 mM, or 2 mM—molarity is fixed for each horizontal row) and hydrogen peroxide (H2O2) (at 20 mM, 100 mM, or 200 mM—molarity is fixed for each vertical column); at six different time points (15, 30, 60, 120, 180, and 360 min). Each reaction condition was quenched using chelex resin (50–100 mesh size). The native HfFucCS and hydrolyzed products from each reaction condition obtained within different time points were analyzed by polyacrylamide gel electrophoresis. Samples (10 μg each) were loaded on a 22% polyacrylamide gel and stained by 0.1% (w/v) toluidine blue (in 1% acetic acid) after adequate electrophoretic migration. The electrophoretic mobility was compared against two molecular markers: LMWH (~8 kDa), and the native HfFucCS (~50 kDa). (TIF) [file pone.0285539.s002.tif]
